# Community focus groups about a COVID-19 individual risk assessment tool: access, understanding and usefulness

**DOI:** 10.1186/s12889-023-16696-3

**Published:** 2023-09-11

**Authors:** Aliyah Keval, Mohammad Titi, Hadi Omar Saleh, Staci Young, Julia Dickson Gomez, Vladimir Atanasov, Bernard Black, John Meurer

**Affiliations:** 1 Medical College of Wisconsin, 8701 W Watertown Plank Rd, Milwaukee, WI 53226 USA; 2 William & Mary, 101 Ukrop Way, Williamsburg, VA 23186 USA; 3 Northwestern University, 633 Clark St, Evanston, IL 60208 USA; 4 Institute for Health and Equity, Medical College of Wisconsin, 8701 W Watertown Plank Rd, Milwaukee, WI 53226 USA

**Keywords:** COVID-19, Risk assessment, COVID-19 vaccines, Focus groups

## Abstract

To make informed COVID-19 related decisions, individuals need information about their personal risks and how those risks may vary with specific demographic and health characteristics. The Fight COVID Milwaukee web-based risk assessment tool allows for assessment of COVID-19 mortality risk as a function of personal and neighborhood characteristics. The purpose of this study is to explore public understanding of this risk assessment tool and risk perception through community focus groups. Individuals were recruited from the general adult population in Milwaukee County, Wisconsin, USA, to participate in nine online focus groups where the risk assessment tool was presented for feedback. Three main themes were identified in the focus groups regarding the web-based risk assessment tool: some challenges in accessibility, variable ease of understanding, and personal usefulness but uncertain value for others. This paper explores how members of the community interpret individual risk assessments and life expectancy estimations, and how these vary with age, gender, race/ethnicity, socioeconomic status, and pre-existing comorbidities.

**Supplementary Information:**

The online version contains supplementary material available at 10.1186/s12889-023-16696-3.

## Introduction

Since its emergence, the coronavirus disease 2019 (COVID-19) has spread rapidly and has had profound effects on the lives and health of people globally. As of July 2023, the WHO reported a total of 768 million confirmed cases globally including 7.0 million deaths and 13 billion vaccine doses administered [1]. The clinical spectrum of COVID-19 infection varies widely from asymptomatic to mild cough, fever, pneumonia, sepsis, respiratory failure, and death. Early detection of patients who are likely to progress to critical illness can aid in delivery of proper care and optimization of limited resources.

Equally important, the COVID-19 pandemic has disproportionately impacted Black, Indigenous, and other people of color (BIPOC) in the United States in terms of increased risk for COVID-19 related hospitalization and death [2–4]. To respond to this and future pandemics, it is essential to have comprehensive knowledge of infection and vaccination rates, the population proportion that will progress to different stages of illness, symptomatic infection, hospitalization, intensive care unit admission and death, and the population proportion that will develop antibody responses. [5] Lack of access to scientifically based knowledge and understandable information may result in avoidable exposure, infection, and illness and to vulnerability to misinformation and unwarranted vaccine hesitancy. Minority, low-income, and elderly populations are particularly vulnerable to these COVID-19 risks.

Policymakers urgently need accurate information about true infection rates and progression risks, along with how those risks vary with demographic and health characteristics in order to make well-informed COVID-19 related decisions. At the individual level, this information is also valuable to determine levels of risk and behavioral precautions necessary to avoid severe illness. Numerous COVID risk assessment tools have been developed in the US as well as abroad [6–8]; however, current approaches to estimating the number of infected persons use statistical models applied to confirmed cases of COVID-19 adjusted to identify possible sources of under detection [9]. The true population infection rate, however, is predicted to be much higher than the infection rate based on viral testing; thus, current assessments will underestimate risk.

Information about risks must be conveyed effectively to the public and especially to vulnerable communities. Fight COVID MKE is a multifaceted research project that utilizes antibody test data, surveys, focus groups and health records from adults living in Milwaukee County to analyze COVID-19 risks from a population perspective. Using these tools, Fight COVID MKE allows researchers to assess risks for hospitalization and death if infected with SARS-CoV-2 and develop and continually update a nationally available web-based individual risk assessment tool. (https://fightcovidmilwaukee.org/individual-risk-estimator).

In this paper, we introduce the Fight COVID MKE individual risk estimator tool, a web-based model that provides individuals with estimates of the risk of dying from COVID-19 for people like themselves, based on various demographic and health-related information. This tool leverages data from Wisconsin, Indiana, and Cook County, Illinois as well as a national 5% Medicare sample of deaths to measure the population proportion infected and how this proportion varies over time, geographically, and with comorbidities. These data calculate the risk that infection for that individual may result in death as a function of individual and community characteristics such as age, gender, race/ethnicity, zip code of residence, body mass index, several chronic health conditions, and importantly, vaccination or past infection status. Surveillance and research are needed to ensure timely understanding of the strengths and weaknesses of vaccines and past infections in preventing illness and death [10]. The risk assessment tool predicts individual risk of life expectancy loss due to COVID depending upon past vaccination and/or infection and community infection prevalence. It compares risk of COVID death and life expectancy loss compared with cancer, car crashes, and pneumonia/influenza. As of July 2023, the risk tool had 2 been used 47,743 times by adults in Milwaukee, Chicago, New York, Los Angeles, and other communities across the US with peak use in January 2022.

The risk assessment tool predicts COVID-19 burden, individual risk to oneself today, and comparison of COVID-19 illness to other common risks. Current literature surrounding COVID-19 risk focuses on public perception of risk and health protective behaviors. However, it is essential to provide a better understanding of the perceptions of risk assessment tools including trust in and understanding of them.

The main aim of this qualitative study was to understand general adult public understanding of the web-based COVID-19 risk assessment tool, to assess their value of using the tool, and to seek their advice how to improve it.

## Methods

### Sampling and recruitment

We developed a protocol and reporting based on key elements of qualitative research [11].

Information letters and flyers were sent to twenty primary care health centers, twelve community and faith partners, and three local health departments around the Milwaukee area with the intent to oversample minority, low-income and elderly populations. Over 900 individuals who participated in the Fight COVID MKE antibody and survey study were also contacted by email to participate in the focus groups. Interested individuals completed a screening survey. Eligibility criteria included being 18 years of age or older, speaking English or Spanish, and being able to provide informed consent. Eligible participants were contacted and informed by research staff of the research purpose, focus group procedure, risks and benefits, and compensation. Voluntary informed consent was obtained from all participants prior to the focus group. The protocol was approved by the Medical College of Wisconsin Human Research Review Board.

Upon agreeing to participate in the study, individuals then completed a questionnaire about COVID illness/vaccination, sociodemographics, household, family, employment, and pre-existing conditions. COVID antibody tests were not done during the focus groups. Participants who indicated interest in participating in focus group interviews were contacted later and scheduled to attend a focus group with age and race/ethnicity matched participants.

Focus groups were completed between July and October 2021 to explore ways participants reduced their risk of exposure to COVID-19 over time and their perceptions of the risk assessment tool. During those four months, Milwaukee County had 25 to 400 new cases of COVID-19 diagnosed daily. During community focus groups, the risk assessment tool was introduced to participants to gauge individual risk perception and obtain feedback regarding accessibility, understanding and usefulness of the tool. In total, 74 individuals participated representing a diverse range of age, gender, race/ethnicities, and social backgrounds (Table 1). Participants were placed into groups of 8–10 individuals based on similarities in age and race/ethnicity. One focus group was conducted in Spanish and the remaining eight in English via Zoom for 90 min by JDG and SY, both experienced qualitative researchers. Other research staff recorded notes during the focus groups.

**Table 1:** Demographics of community FCM focus groups

Characteristics	n(%)
Gender	
Male	24 (32%)
Female	47 (64&)
Other	3 (4%)
Age	
20-35	18 (24%)
36-50	22 (30%)
51-65	18 (24%)
65+	16 (22%)
Race/Ethnicity	
Non-Hispanic White	27 (36%)
Hispanic White	21 (28%)
Black/African American	19 (26%)
Asian	4 (5%)
Other	3 (4%)

### Focus group discussion content

Based on literature review and research team interests, focus group discussions concentrated on participants’ general feelings about medical research and what would encourage their participation, their understanding of COVID-19 antibodies, behavioral changes in their lives due to COVID-19 including social distancing, childcare, food security, housing, experiences with COVID-19 illness, and trust in the COVID-19 vaccine. Additionally, focus group facilitators presented different ways of communicating community and individual risk to COVID-19 through the Fight COVID MKE risk assessment tool. Participants were asked to react to the risk assessment tool to determine the most understandable and acceptable way of communicating risk.

### Analysis

All focus groups were audio recorded and transcribed verbatim. Analysis was conducted by multiple researchers using a qualitative data analysis tool MAXQDA. Codes were utilized to identify positive and negative perceptions of the risk assessment tool, including ease of accessibility, understanding, and perceived usefulness. Initial codes were developed and refined collaboratively among the research team using an iterative process. Discrepancies in code labels were discussed in team meetings and a consensus was reached among all members. The team then identified general themes representing data across all focus groups. Analysis continued until no new significant themes emerged. We reached saturation of themes with 74 participants.

## Results

Thematic analysis of the Fight COVID MKE focus groups revealed three main themes regarding the web-based risk assessment tool: (1) Ease of understanding; (2) Tool utility; and (3) Accessibility. Each theme discussed in the focus groups contained positive and negative perceptions that give insights to how community members perceive and may be influenced by risk assessment tools. Feedback was addressed by risk tool developers after the first four focus groups; thus, later focus groups responded to a revised version of the risk assessment tool with recommended improvements including changing from tables of numbers to charts and figures depicting the information and a video explaining use of the tool and interpretation of the results.

### Ease of understanding

Focus group participants commented on the ease of understanding information presented in the risk assessment tool, with some participants finding the information helpful and easy to understand overall. One participant (Mixed-race female, 53 years of age – Focus Group (FG)8) had used the tool prior to their focus group session and noted the tool was “informative and interesting to see”. Other participants that were able to understand the tool spoke to its simplicity and being able to digest the information presented:

*“This is very easy to navigate so far that I can see. Comprehensive, and it doesn’t really – it’s not too arcane. So, pretty, pretty easy – pretty easy to navigate, for me, for sure.” (Mixed race male, 29 years of age - FG8)*

Specific aspects of the risk assessment tool that participants found to be especially straightforward included components that provided a visual representation of risk, which participants generally found easier to follow and digest than words and numerical explanations. One of these visual representations is a bar graph that compares the risk of death due to COVID-19 in individuals who were (a) not previously vaccinated or naturally infected with COVID-19 to individuals who (b) are maximally protected by way of vaccination and/or previous infection:

*“I think the graph itself is good because that shows you the comparison of individuals that are vaccinated vs. unvaccinated. So, I think that could be a powerful tool in encouraging people to consider getting vaccinated. (White female, 73 years of age - FG4)*

*“The black box with the yellow dots and the blue dots, well, that’s pretty clear to me that if you get vaccinated, look where you’ll be compared to if you don’t. That one I like.” (White female, 77 years of age - FG1)*

Other participants felt the tool was not easy to understand. One participant described the tool as being “too complicated” (White female, 68 years of age—FG4). Others felt similarly and spoke of how they had difficulties interpreting information and grasping what the tool was conveying:

*“I don’t know that people would look at this and realize it’s an assessment to figure out their COVID risk.” (White male, 74 years of age - FG1)*

*“I think many of us are saying that it’s a bit unhelpful because there are too many unexplained categories.” (White female, 73 years of age—FG4)*

One specific source of confusion for users of the tool in the focus groups pertained to the component that compares life expectancy or loss of life due to COVID-19 to other common causes of death including cancer, flu/pneumonia, and motor vehicle accidents:

*“I think this black box, “COVID burden for people like you,” and the life expectancy from other causes, I don’t think people are going to relate to this, or understand how it relates to the information that they put in above.” (White female, 75 years of age - FG1)*

*“I mean, people don’t know what that means… What does that mean? Maybe other people think, “Wow, that’s really interesting.” But most people would say, “Well, I don’t even know what that means.” It means I live three days longer, or what does that mean? I don’t think people understand.” (White female, 68 years of age - FG4)*

In response to these perceptions of specific components of the risk assessment tool, the lead researchers used feedback from initial focus groups to translate these assessments into further improvements of the tool. These changes primarily consisted of changing figures estimating risk by vaccination status to bar graphs instead of numerical data in table format. All subsequent changes made to the tool were updates with COVID mortality data and vaccine effectiveness. Researchers aimed to ease understanding of each component addressed in the focus groups to reach broader populations and facilitate COVID-19 risk perception among those who use the tool. Additionally, the research team uploaded a tutorial video to the webpage explaining the purpose of the risk assessment tool, how to use it, and how to enter and interpret personal data. Five additional Zoom focus groups were held following the addition of the tutorial and the video was played during the subsequent focus groups prior to discussing feedback. Consequently, participants generally found the video aided in their understanding of the risk assessment tool:

*“The way [the video] just explained makes perfect sense, how the numbers should be interpreted and stuff. But when I was looking at an actual page, just doing it myself – for example, the category that is meant to say how much time would be taken off of your life if you had COVID, I interpreted that, I think, as that's how long you'd have left to life if you got COVID or something like that.” (White gender non-conforming, 26 years of age - FG6)*

### Tool usefulness

The goal of creating a web-based COVID-19 individual risk assessment tool such as this one is not to provide medical advice, but rather to allow individuals the ability to determine individual risk, consider the impact of vaccination, and, as one participant noted, disseminate information that is “data-driven” (Native American male, 36 years of age—FG8). Focus group participants who found the risk assessment tool to be helpful and informative further elaborated on the utility of the tool, specifically discussing the impact that this tool could have on individuals who may not otherwise know how to evaluate their risk:

*“I think this is a great tool. I’m playing around with it. I’m seeing the impact that my age, my race, my zip code kind of has on the risk of infection. I think it’s very interesting to see.” (Mixed-race female, 28 years of age - FG8)*

*“I’m certainly gonna suggest it to my group of non-vaccinated family members and friends. I’m gonna do whatever I can to push them toward that.” (African American female, 55 years of age - FG2)*

However, participants noted that although they may have found the tool to be personally useful and would recommend it to others in their communities, this may not hold true or connect with certain populations who harbor negative attitudes with respect to the pandemic and vaccination. While discussing these pervasive negative societal attitudes, many noted that individuals who may not be vaccinated or engaging in protective behaviors “don’t trust the information that’s out there” (White female, 67 years of age—FG1) or remain “skeptical” (White male, 70 years of age- FG4) of the foundational science on which the tool is based. Many had concerns about the political climate surrounding the COVID-19 pandemic:

*“I think we’re all thinking the same thing that in this very hyper-politicized climate that we’re all living in right now, there’s a lot of people who feel like it doesn’t matter what you say to them, you know, it’s fake news, it’s misinformation, it’s not real science.” (Mixed-race male, 29 years of age - FG8)*

Likewise, some participants discussed how the impact of the risk-assessment tool is ultimately up to individual interpretation and may result in “counterproductive” (White male, 70 years of age—FG4) use. In this thought process, participants examined how a young, vaccinated or unvaccinated individual’s results that show a fairly low risk for severe illness and death from COVID-19 infection may convey a false sense of security and therefore result in lower risk protective behaviors:

*“I would be interested to know what the likelihood of someone changing their vaccine plan would be based on seeing this data if it would help anyone to see the value of getting the vaccine or if this would maybe embolden some younger folks to say, well, you know, it's not that big of a deal, look at the risk of me dying, it's fine.” (White female, 45 years of age - FG5)*

Participants who initially struggled with understanding the purpose of the risk assessment tool and the information it conveyed also displayed doubt and distrust of the tool for use within the public. These participants stated that the information disseminated in the tool seemed largely intended for the academic community and one participant noted that “You’re wasting your time trying to convince the average lay person to get vaccinated with this [tool].” (White male, 74 years of age—FG1).

Others noted that their difficulty in interpreting the results from use of the tool would make it less likely for them to disseminate the information within their own social circles. A few participants questioned how useful this information that may come across as overwhelming will be to the public, especially those who remain unvaccinated due to distrust:

*“This is a little bit overwhelming, all of this stuff, for the average lay person, to listen to all of this. There’s got to be a better way to get to people that are not vaccinated than this. Because they’re not going to listen. Frankly, I don’t want to listen. I’m sorry to say that, but that’s just the way I feel.” (White male, 74 years of age - FG1)*

### Accessibility

When creating a public, web-based tool, it is essential to consider the accessibility and perceived barriers towards using the tool. Some confusion was evident throughout multiple focus groups about the logistics of the tool—how it would be accessible to the public, if its use was limited to healthcare settings, or only to those participating in the Fight COVID MKE study. The research team aimed to communicate in both the focus groups and within the tool web page itself that it is a tool meant to be used by any adult nationwide.

Some participants were unsure whether the tool needed to be used in the presence of their physician who could explain the results. This was noted most often in early focus groups that responded to a tool that was deemed less comprehensible. Participants felt that in order to make a tool like this nationally available and useful to the public, it must be digestible for a layperson with little or no medical background. Additionally, they wondered if all adults would have internet access to the tool. Participants were also concerned about whether some members of the public understood a very important aspect of the tool – chronic health conditions or comorbidities:

*“You’re assuming that they know if they have any of these diseases that it increases their risk of COVID.” (White female, 75 years of age - FG1)*

Participants pointed out that some people with less adequate or equitable access to healthcare would also be less likely to have been diagnosed with comorbidities. In order to interpret one’s individual risk with regards to COVID-19 infection, there is some assumption with the tool that people understand why their risk may be elevated or not:

*“Until I got a decent job, I probably couldn’t answer these questions because I couldn’t afford to go to a doctor. So, how could I say I had a chronic illness?” (African American male, 51 years of age - FG2)*

## Discussion

Results from this study suggest that although many focus group participants found the risk assessment tool potentially useful, its usefulness was directly linked to the level of ease in understanding it. Understanding the tool was related to participants’ limited numeracy and health literacy. Although some participants were able to interpret their personal risk with the tool, others struggled to completely grasp this information and expressed their lack of understanding. Limited numeracy led to insufficient comprehension in assessing risk and is common within the general population, specifically as it pertains to the interpretation of health-related statistical concepts [12]. Consequently, this makes communicating health risk statistics and similar probabilities more difficult as individuals may be less capable of interpreting the information and acting upon it to construct well-informed self-care and medical decisions. Additionally, vulnerable groups with impaired access to healthcare, as was sampled in this study, have lower rates of numeracy along with other aspects of health literacy [13]. Therefore, when communicating health risks, it is essential to present this information using simple graphs, charts, and video guides describing how to use and interpret data.

Additionally, medical mistrust and prior perceptions of healthcare serve as barriers to individual willingness to adopt recommendations provided by researchers. Medical mistrust has been well documented among ethnically/racially underrepresented communities historically [14] but has been recently more widespread, even in majority populations, due to increased levels of misinformation regarding the COVID-19 pandemic [15]. This mistrust in healthcare translates to mistrust of scientific data as well, presenting a challenge in using data to convince those who do not believe in science. Previous studies have shown that medical mistrust and misconceptions are associated with decreased adherence to health recommendations and as a result, individuals with these beliefs are less likely to engage in COVID-19 specific health protective behaviors [15–17]. Decreasing these barriers can address a limitation in the utility of the risk assessment tool among certain populations, especially among ethnic and racial minorities.

Limitations of this study included the small sample size and recruitment of a convenience sample of community members throughout Milwaukee County. In the participant screening process, an emphasis was placed upon sampling vulnerable members of the urban community to explore their perceptions of risk assessment. As such, the viewpoints of these participants may not reflect the views of members in other communities, such as those in rural or suburban areas. Additionally, the perceptions explored in the smaller sample size within this study may not be generalizable to the public; however, the qualitative approach used allows for the real-time navigation of and analysis of individual perceptions of risk assessment tools and continual improvement of these instruments. Given that focus groups were conducted online via Zoom, participation was limited to those with access to the internet and audio dial-in capabilities. Lastly, participation inequality existed in the focus group sessions as a few active individuals offered more to the discussion on risk assessment methods compared to other participants.

## Conclusion

Results from the evaluation of this risk assessment tool are some of the first to disseminate data regarding ways in which community members perceived and responded to a public web-based COVID-19 risk assessment tool. This tool provides both individuals and healthcare professionals with pertinent information geared toward individual COVID-19 risk factors. Focus group discussions centered around ease of understanding, tool utility, and accessibility. The themes discussed in this paper explore how members of the community perceive individual assessments of risk, estimates of life expectancy, and how they vary with age, gender, race/ethnicity, socioeconomic status, and pre-existing comorbidities. Because risk assessment involves identifying and understanding multiple variables, developing a comprehensive, digestible assessment is a challenging task. Notwithstanding, a successful risk assessment tool could be a valuable instrument in educating community members and changing their behaviors to adopt healthier lifestyles.

### Future implications

Understanding COVID-19 infection and progression rates, and how they vary with a full set of patient-specific characteristics is important for effective policy and practice responses to the COVID-19 pandemic as well as for future infection outbreaks. Health, demographic, mortality, and other data is essential for obtaining unbiased estimates of these rates. The Fight COVID MKE risk assessment tool provides adults with access to information about how the COVID-19 pandemic has impacted individuals with similar personal characteristics to guide in assessing their individual risks. Additionally, the empirical methods developed for the risk assessment tool might be used and modeled for other infection outbreaks in the future.

### Supplementary Information

**Additional file 1.**

## Data Availability

The datasets and codes that support the results of this study are not available from the corresponding author because the participants did not provide consent for release of the data.
