# Supplementary material for: Community focus groups about a COVID-19 individual risk assessment tool: access, understanding and usefulness
Source: BMC Public Health. 2023 Sep 11;23:1761. doi: 10.1186/s12889-023-16696-3 (PMC10494421; doi:10.1186/s12889-023-16696-3)
Supplement: Supplementary file 1 — Additional file 1. [file 12889_2023_16696_MOESM1_ESM.docx]

**Fight COVID Milwaukee community focus group questions**

**Screening questions for community participants asked of individuals by research coordinators prior to the focus group:**

1. What is your race/ethnicity?
   - White non-Hispanic
   - Black/African American
   - Latino/Hispanic
   - Other
2. What is your gender?
3. What is your age?
4. Are you currently working? If yes, what is your occupation?

- If a participant answers store cashier or stock person, waiter, construction worker, bus driver, first responder (EMS, fire, police), health care professional with direct contact with patients, consider them as in a high-risk job

1. Do you have children living at home with you? What are the ages of your children?
2. Were you infected with COVID?
3. Did you receive a positive COVID-19 test?
4. Did you become severely ill or were you hospitalized with COVID-19?
5. Do you have a pre-existing condition that makes you more vulnerable to COVID? What is that condition? Consider the following to be people with preexisting conditions: diabetes, COPD, smoking, cancer treatment, HIV, obesity, heart disease or high blood pressure.

**Focus group questions for community participants in semi-structured format that may be edited slightly during a group:**

**Perspectives on research participation:**

1. What is a research study for health? What experiences have you had, if any, participating in a research study? What were some positive aspects? What were negative aspects? Do you feel like you understood the purpose of the research? In what ways did you feel you understood research? In what ways did you not understand? Did you feel like you understood what would happen to you in the research project? What aspects, if any, did you not understand? How could researchers have better communicated with you?
2. What kinds of things would make you trust a research project enough to want to participate?

**Personal experiences with COVID-19:**

1. During COVID-19, what kinds of things did you do to keep yourself safe? How reasonable did you think that public health recommendations for masks and social distancing were? Were you able to follow them? Why or why not?
2. Did you or anyone you know get sick with COVID? What were your experiences or the experiences of other people you know? For example, did they become seriously ill? Was there any delay in getting diagnosed? Were you or they hospitalized? Do you know anyone who died from COVID?
3. How much contact did you have with people who became ill? What were the circumstances in coming to contact with them?
4. How comfortable did you feel going to the doctor during the pandemic? What did you weigh in deciding whether or not to seek regular medical attention (i.e., not for suspected COVID) from a doctor?
5. How comfortable were you in taking public transportation? Could you avoid taking public transportation? Why or why not?
6. How did the transition to virtual learning affect you and/or your children’s learning? You and your children’s emotional health? Your ability to work?
7. How did the childcare closures affect you?
8. In what other ways were you affected by COVID-19?

**COVID-19 and perceptions of risk:**

1. Our project hopes to find out what things put people at greater risk for becoming infected with COVID-19. What are antibodies?  Why are they important to health?
2. What are the best ways to communicate individual health risks? Community health risks? What does high risk mean to you? What does low risk? How would you assess a 1 in a thousand risk? 1 in a hundred? 1 in 50?
3. What are your concerns about influenza vaccines? COVID vaccines? How can those concerns be reduced?

**FCM risk assessment tool:**

1. Overall, how useful do you find this tool? What could make it better? More understandable?
2. How do you understand the following?
   - Your life expectancy – how long someone like you will live, on average.
   - The proportion of people like you who have died from COVID.
   - How much COVID has reduced life expectancy for people like you.
3. Take a look at the box with the difference in life expectancy after vaccination. What does this mean to you? How do you understand the difference in life expectancy after becoming infected?
4. Do you believe these estimates? Why or why not?
5. Looking at the bottom box, how useful is the comparison with loss of life expectancy from motor vehicle crashes, cancer, or flu or pneumonia?
